# A Guide to Best Practice in Sensory Analysis of Pharmaceutical Formulations †

**DOI:** 10.3390/pharmaceutics15092319

**Published:** 2023-09-14

**Authors:** David Clapham, Emilie Belissa, Sabine Inghelbrecht, Anne-Marie Pensé-Lhéritier, Fabrice Ruiz, Liz Sheehan, Margaret Shine, Thibault Vallet, Jennifer Walsh, Catherine Tuleu

**Affiliations:** 1 Independent Researcher, Bishop’s Stortford CM23 4FQ, UK; 2 UCB Pharma, 1420 Braine l’Alleud, Belgium; emilie.belissa@ucb.com; 3 Janssen Research & Development, Johnson& Johnson, 2340 Beerse, Belgium; singhelb@its.jnj.com; 4 FRM GaleSens, 95000 Cergy, France; annemarielheritier@gmail.com; 5 ClinSearch, 92240 Malakoff, France; fabrice.ruiz@clinsearch.net (F.R.); thibault.vallet@clinsearch.net (T.V.); 6 SRL Pharma, T12 XF62 Cork, Ireland; lsheehan@srlresearch.com (L.S.); mshine@srlresearch.com (M.S.); 7 Jenny Walsh Consulting Ltd., Nottingham NG2 1EP, UK; jenny@jennywalshconsulting.com; 8 UCL School of Pharmacy, London WC1N 1AX, UK

**Keywords:** palatability, acceptability, taste, mouthfeel, sensory analysis, sensory data, non-human sensory analysis, human sensory analysis, risk assessment, compliance

## Abstract

It is well established that treatment regime compliance is linked to the acceptability of a pharmaceutical formulation, and hence also to therapeutic outcomes. To that end, acceptability must be assessed during the development of all pharmaceutical products and especially for those intended for paediatric patients. Although acceptability is a multifaceted concept, poor sensory characteristics often contribute to poor patient acceptability. In particular, poor taste is often cited as a major reason for many patients, especially children, to refuse to take their medicine. It is thus important to understand and, as far as possible, optimise the sensory characteristics and, in particular, the taste/flavour/mouthfeel of the formulation throughout the development of the product. Sensory analysis has been widely practiced, providing objective data concerning the sensory aspects of food and cosmetic products. In this paper, we present proposals concerning how the well-established principles of sensory analysis can best be applied to pharmaceutical product development, allowing objective, scientifically valid, sensory data to be obtained safely. We briefly discuss methodologies that may be helpful in reducing the number of samples that may need to be assessed by human volunteers. However, it is only possible to be sure whether or not the sensory characteristics of a pharmaceutical product are non-aversive to potential users by undertaking sensory assessments in human volunteers. Testing is also required during formulation assessment and to ensure that the sensory characteristics remain acceptable throughout the product shelf life. We provide a risk assessment procedure to aid developers to define where studies are low risk, the results of a survey of European regulators on their views concerning such studies, and detailed guidance concerning the types of sensory studies that can be undertaken at each phase of product development, along with guidance about the practicalities of performing such sensory studies. We hope that this guidance will also lead to the development of internationally agreed standards between industry and regulators concerning how these aspects should be measured and assessed throughout the development process and when writing and evaluating regulatory submissions. Finally, we hope that the guidance herein will help formulators as they seek to develop better medicines for all patients and, in particular, paediatric patients.

## 1. Introduction

It is well established that treatment regime compliance is linked to the acceptability of a pharmaceutical formulation, and hence also to therapeutic outcomes [1,2,3,4]. It can clearly be stated that medicines cannot work unless they are correctly used by the patient during the complete treatment period [1]. Children are, on the whole, more likely to find a product less acceptable than an adult [5,6,7].

To that end, acceptability must be assessed during the development of a paediatric product and acceptability evaluation should be included in the studies outlined in Paediatric Investigation Plans (PIPs) and Paediatric Study Plans (PSPs) [1,8].

Although acceptability, defined by the EMA as the overall ability and willingness of the patient to use, and its care giver to administer, the medicine as intended, is a multifaceted concept [9], several of these aspects relate to how the product is perceived by the senses of the user. These include its appearance (e.g., colour, size, shape), usability, skin feel/texture of a topical product, and palatability. Palatability is defined as the overall appreciation of a medicinal product (often oral) in relation to its smell, taste, aftertaste, and texture (i.e., feeling in the mouth). Poor sensory characteristics often contribute to poor patient acceptability. In particular, poor taste is often cited as a major reason for children to refuse to take their medicine [10,11,12].

It is thus important to understand and, as far as possible, to optimise the sensory characteristics of the formulation throughout the development of the product.

Despite the fact that the importance of achieving acceptable sensory characteristics is well recognised and agreed upon by regulators, formulators, patients, and their caregivers, there is confusion about how best to measure these aspects, how to optimise them, and how to track potential changes during product storage.

The European Paediatric Formulation Initiative (EuPFI; www.eupfi.org, accessed on 8 September 2023) was founded in 2007 and is a consortium of over 20 institutions working in a pre-competitive way on paediatric drug formulations. The EuPFI Taste Assessment and Taste Masking (TATM) workstream has assembled a team of experts to use their knowledge and experience to offer guidance on best practices that may be employed when assessing the sensory aspects of medicinal products.

Even though sensory aspects of a product are inherently subjective, with proper experimental design and careful control, objective information can be derived from sensory studies. Sensory analysis has been widely practiced for food and cosmetic product development and assessment. Here we present proposals concerning how the well-established principles of sensory analysis can best be applied to pharmaceutical product development. We hope that this guidance will also lead to the development of internationally agreed standards between industry and regulators concerning how these aspects should be measured and assessed throughout the development process and when writing and evaluating regulatory submissions. Finally, we hope that the guidance herein will help formulators as they seek to develop better medicines for all patients and, in particular, paediatric patients.

## 2. Useful Non-Sensory Analytical Data

In some cases, a combination of an understanding of the mechanism of sensory perception and standard analytical techniques can be helpful. These include the following:

### 2.1. Dissolution

To elicit a sensory response, the active pharmaceutical ingredient (API) must be present at the taste receptors at a concentration above its taste detection threshold. Any formulation or taste masking method that keeps the concentration of API in the mouth below that threshold throughout the dose delivery time fundamentally cannot be aversive due to the API itself [13]. Typical examples of such taste masking methods are physical or chemical barriers to drug release in the mouth such as complexation or coating [14,15], or use of a salt/version or form of the API that has low solubility in the mouth [16].

API concentration in the mouth may be demonstrated using a suitable dissolution method with an appropriate dissolution medium such as artificial saliva [17]. Thus, it should be possible to screen candidate formulations for those more likely to have acceptable taste.

However, lack of bitterness or other unacceptable taste of the API alone may not produce an acceptable formulation. The drug product may have other undesirable characteristics such as other aversive taste (i.e., metallic, burning, etc.), poor smell, or mouthfeel (such as grittiness) related to the API or excipients. Therefore, a human sensory study may still be required for final selection of the optimal formulation.

### 2.2. Rheology/Texture Analysis

Rheological characteristics/textural analysis can be linked with the mouthfeel of oral products and, in particular, with the skin feel of topical products using the techniques of tribology [18,19,20]. It is unlikely that rheological assessment alone will permit assurance that a new product will have an acceptable mouthfeel/skin feel. However, formulations can be screened for changes in these aspects over time/during storage. Where changes in rheological profile/texture have not occurred for a formulation that has previously been demonstrated to be acceptable, it is reasonable to assume that the product remains acceptable. Where changes have occurred, there may be a need for a human study to investigate whether the product remains acceptable. If the change observed is large, it may be possible to conclude that the formulation will not be acceptable based on the rheology/texture data alone.

Although these methods can be helpful in establishing the long-term stability of a formulation that has previously been demonstrated to be acceptable, and may have some utility in screening prototype formulations, they generally have limited utility in proving the overall acceptability/palatability of a new formulation. For that, a more integrated approach is required.

## 3. Sensory Data from Non-Human Studies

Since only a human consumer can provide reliable information about whether or not a product is acceptable to them, any information gained from non-human sensory studies needs to be validated against data derived from human responses or be based on a fundamental understanding of sensory science. Nevertheless, in some instances, non-human sensory data can act as a screening tool and/or provide supporting data to aid the product developer in their formulation strategy and help them in reducing the number of times that samples need to be assessed by a human panel, thus reducing the overall number of samples that need to be tested by the panel. Consideration should therefore be given to whether any of these methods could be used to reduce or eliminate the need to expose a human volunteer assessor to the product.

The applicability of various taste assessment methodologies was discussed in a previous EuPFI review [21]. This paper concluded that whilst several methods offered some potential for assessing the aversiveness of an API alone, and hence possibly aiding the selection of molecules that would be easier to formulate into an acceptable product, only two showed promise for aiding in formulation development and/or assessing the stability of the taste aspects of a formulation. These were the electronic tongue and the rodent brief-access taste aversion (BATA) models. Data from the e-tongue may also be combined with data from an e-nose to potentially provide a fuller understanding of the flavour of a product.

### 3.1. Electronic Tongue

A range of devices that come under the umbrella term “electronic tongue” have been developed or proposed [22,23,24]. They can in principle provide an initial indication of the approximate taste intensity (usually bitterness) of an API and information about certain other taste properties, depending on the device, sensors, and calibration model used.

All current e-tongues utilise a sensor array where the sensors are semi-selective for one or more of the basic taste modalities (bitter, sweet, sour, salty, umami). The individual sensors respond to different groups of dissolved organic and/or inorganic compounds. That being so, the signal is dependent on the solubility of the API and other formulation components. The “fingerprint” potentiometric output from a set of these sensors can then sometimes be used to build a model that correlates with human taste responses, either (and preferably) to that of the individual API being formulated, a comparator molecule, or from a calibration set of other molecules, using chemometric methods and visualisation on a two- or three-dimensional plot.

The two e-tongues that have received the most attention in the pharmaceutical sphere are the Alpha Astree (Alpha-MOS [25]) and the Insent (Intelligent sensor technology [26]) devices.

E-tongues have several potential uses in product development/sensory assessment, as described below.

#### 3.1.1. Molecule Selection

At the earliest stage of product development, it may be possible to use an e-tongue calibrated with a range of molecules of known bitterness to provide an indication of the probable bitterness of a new molecule [27]. There is however a risk that the response from the new molecule falls outside the range of applicability of the calibration model.

#### 3.1.2. Taste Masking

It is possible to screen potential taste masking options using this technology. For example, this may be achieved by comparing the distance between the unmasked API, a placebo, and the potential taste masked formulations using the multidimensional distance between them. The smaller the distance between the “masked” product and the placebo, the more similar will be the taste of each formulation compared to the other. It is important to recognise that for this to be true, all other aspects, such as pH and ionic strength, remain the same between the two formulations, as these aspects can also affect the response of the sensors.

There are several successful applications of this approach reported in the literature, (e.g., [28,29,30,31]), allowing the best of a range of possibilities to be selected for further evaluation. However, the e-tongue results cannot be relied upon to show that even the best of the formulations selected has sufficient acceptability, and all data must be validated against assessment by a human panel.

#### 3.1.3. Stability Assessment

The sensors used for e-tongues have some inherent variability over time, so reliable comparisons require that samples be assessed side-by-side on the same instrument within a short timeframe even if correction algorithms are used [32]. Nevertheless, if a control sample of the formulation that is known not to change in its taste characteristics is available (for example, a sample kept under refrigeration), then a sample drawn from a stability study can be compared with it. If the position of the control sample and the test sample appear in the same area of the chemometric plot within an acceptable tolerance, they can reasonably be concluded to have the same taste. If this is not the case, then something has changed. This may be a change in taste, pH, or another factor. Whether the change is acceptable to the patient or not can only be established by human sensory analysis.

### 3.2. Electronic Nose

The overall palatability of a formulation is a combination of the base taste detected by the tongue and the volatile components from the formulation, and, in particular, the flavour used, that are detected by the nose. Many e-nose devices have been developed that utilise sensor arrays to “fingerprint” the headspace above a sample, again using chemometrics, which may then be correlated with the human sensory response.

Widely used for some sensory applications in the food and beverage industries (e.g., [24]) the data from an e-nose can be helpful alone, or in combination with the data from an e-tongue, in monitoring changes in the formulation over time [33,34]. Nevertheless, e-nose data cannot help in molecule selection and are unlikely to be of use in taste masking assessment.

### 3.3. Rodent Brief-Access Taste Aversion BATA Model

The rodent BATA model may be regarded as the most frequently used of a range of whole animal methods for assessing the overall aversion/pleasantness of a pharmaceutical product. The rat is the rodent most frequently used in pharmaceutical studies.

The assessment is made by exposing a mildly water-deprived rodent to a 100% acceptable standard (water), an aversive calibrant at various concentrations (usually quinine or caffeine), and the test substance and/or its formulations. The willingness of the rodent to taste the sample, measured as the number of times they will lick at a spout or a well containing the sample during a pre-defined sample period, is counted electronically.

Water provides a “fully acceptable” standard, whilst the bitter calibrant provides a calibration curve of aversiveness. The test samples can then be compared with this calibration curve and their relative acceptability established.

Since the model shows excellent correlation with adult human responses [24,35], it is possible to establish whether or not a test formulation is likely to be adequately acceptable to the average human volunteer assessor in terms of taste [36].

However, despite the good correlation observed to date between rat and human data, there is the possibility that this may not be the case for some APIs. In addition, as described above, acceptability is a multisensory and multifactorial concept. Therefore, results generated from the rodent BATA model or another in vivo assessment using a different animal model will likely need confirmation or validation using human subjects [37].

The responses from a survey of regulators from across Europe (see Section 4.1.2 and Q9 Appendix A) indicate that non-human sensory data alone would not be acceptable as sole evidence of the palatability of a formulation, and in some cases would not even be acceptable supporting evidence.

### 3.4. Colour/Appearance

The colour and other appearance aspects of a product can be measured more or less objectively using, for example, EP/USP [38,39] pharmacopeial colour standards, Munsell Chips [40], tristimulus colorimetry [38,41,42,43], turbidity/nephelometry assessment [44,45,46], and gloss measurement (goniometry) [47,48,49]. It may be possible to use these data to formulate a product with characteristics that match those of a product that is already known to be acceptable. Human assessment of continuing acceptability will otherwise only be required if a significant change is detected or the product is novel in terms of its appearance.

Given that colour data can be obtained using pharmacopeial methods, there is no need to provide data from human subjects, although this may be required in terms of the effect of the appearance on the overall acceptability of a product.

## 4. Human Sensory Analysis

**Figure 1:**
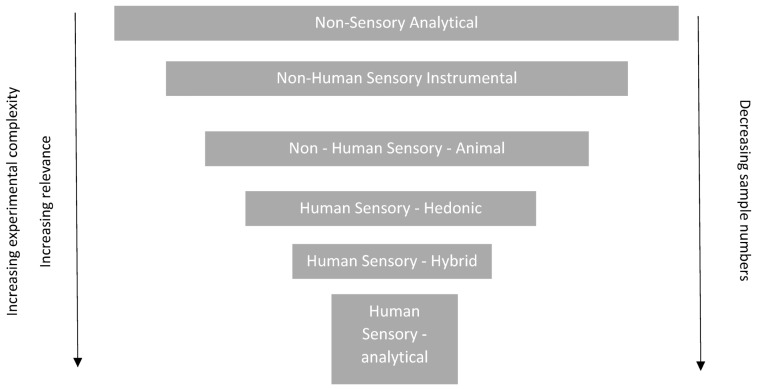
An insight into the challenges and benefits of the various approaches.

Although it may be desirable to reduce the number of taste assessments using human volunteers, by employing some of the methods discussed in Section 1 and Section 2 of this paper, these will rarely be sufficient on their own. Figure 1 Gives an overview of challenges of various approaches. In the end, the acceptability of the sensory characteristics of a pharmaceutical product to a patient, which will help to ensure compliance/adherence with treatment regimes, can only be confirmed by testing it in a human, and preferably in the target patient population, since taste perception can be affected by age, sex, and disease state, amongst other factors.

For veterinary medicines, guidelines exist to aid the formulator to assess the palatability of a proposed product in animals [50]. In contrast, at present, no such regulatory guidance exists for assessing the sensory characteristics and acceptability of pharmaceutical products intended for humans, whether adult, geriatric, or paediatric.

In this section of the paper, we provide some guidance based on experience of applying the well-established principles of sensory analysis, using humans as the testing instruments, to pharmaceutical product development/evaluation at various stages of the pharmaceutical development process.

Sensory science is a well-established discipline widely practiced in food and cosmetic product development and assessment (e.g., [51]), but is less well known or understood in the pharmaceutical sphere. While it is universally recognised that sensory characteristics of products are inherently subjective, with the use of proper experimental design and carefully controlled sensory studies, useful objective information can also be derived. Therefore, during the pharmaceutical development process, not only can subjective information such as acceptability and palatability be measured, but analytical measurements of sensory characteristics can also be made.

While the principles outlined below are generally applicable to the evaluation of any sensory aspect of a formulation (or any sensory modality of a product), it is for the assessment/evaluation/measurement of taste/palatability, in particular bitter taste, that the use of human sensory studies are invaluable and, in many cases, absolutely necessary. This is for three main reasons.

Firstly, it is well established that for formulations where the API can interact with the taste buds in the oral cavity (such as oral liquid dosage forms (solutions, suspensions, emulsions), oral films, dispersible tablets, buccal tablets, lozenges, oral powder sachets, uncoated tablets, inhalers, etc.), taste is either the major factor, or at least one of the major determinants, of product acceptability and patient compliance with treatment [10,11,12]. This is especially true in paediatrics because children are more sensitive than adults to bitterness which, unlike sweet flavours, is naturally aversive [52,53,54]. Secondly, the oral route is the most commonly used dose delivery route [55]. Thirdly, assessment of palatability, unless correctly conducted, has the highest potential to expose the volunteer (also known as the volunteer assessor) to ingestion of the API.

**Figure 2:**
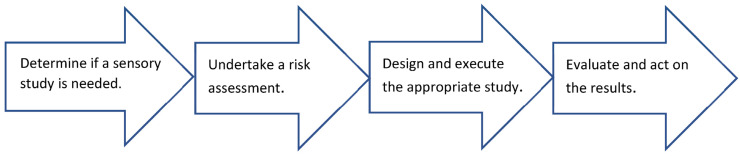
Human sensory study flow diagram.

To this end, the following basic flow (Figure 2) should be followed for all sensory studies where a human volunteer assessor evaluates the sensory attributes of a product containing an API.

The remainder of this paper provides more detailed guidance for each of the above broad steps to provide a tool for readers to undertake such sensory studies. In a second paper, we will provide examples of how the approach may be applied to various stages of product development.

### 4.1. Risk Assessment

#### 4.1.1. Introduction

Product development requires the ability to test candidate formulations during development in order to select the best candidates and then ensure that they remain organoleptically acceptable throughout their shelf life. As already stated, this will often require assessment by a human.

Most sensory studies undertaken in the development process require that the API is present in the formulation, although it may sometimes be possible to use a placebo or a surrogate molecule in place of the particular API. If the API is present, then there is a potential risk of absorption of the active substance even if a “rinse and spit” (also known as “swirl and spit” or “sip and expectorate”) design is used. However, with a well-thought-out evaluation of the challenges posed by each API and the correct study procedure, this risk can be minimised and/or mitigated. Balanced against this risk is the need to generate sensory data to develop an acceptable product which will benefit patients, and which is a requirement for paediatric products.

A properly conducted risk assessment will ensure the safety and wellbeing of the volunteer assessors, define the environment in which the study may be undertaken, and inform the study design.

#### 4.1.2. Study Type

The first step in the risk assessment (after confirming that a human sensory assessment is needed) is to define the study type/environment. A key decision that needs to be made is whether the study MUST be conducted as part of a clinical trial, whether as part of an existing planned trial or one designed specifically to obtain sensory data, or whether it can safely be conducted as a standalone sensory assessment. Currently there appears to be some significant confusion within industry on this point. To attempt to provide some clarity, we provide a table below (Table 1) outlining some of the benefits and challenges of conducting studies in each of these environments.

We also conducted a 10-question survey among various regulatory agencies to seek to understand their thinking on palatability/sensory studies. The results are summarised in Appendix A and referenced where relevant throughout this document.

In summary it appears that standalone sensory studies are permitted in many jurisdictions, though perhaps not in others, provided that they are assessed as low risk and have ethical approval, since they are not formally regarded as clinical trials [56,57] (see Q1). As such, they mostly do not need to be notified of sensory studies (Q2–Q4). The same thinking applies regardless of the type of sensory study (Q8).

Q6. Interestingly the agencies were roughly evenly split on whether data from adult panels would be considered sufficient for establishing acceptability in a paediatric population. However, they reported that this may be accepted with justification, either alone or as supportive evidence, along with data from the target population. Ideally, all authorities would like to see data from the target population in the drug development dossier.

#### 4.1.3. Risk Assessment Flow Chart

**Figure 3:**
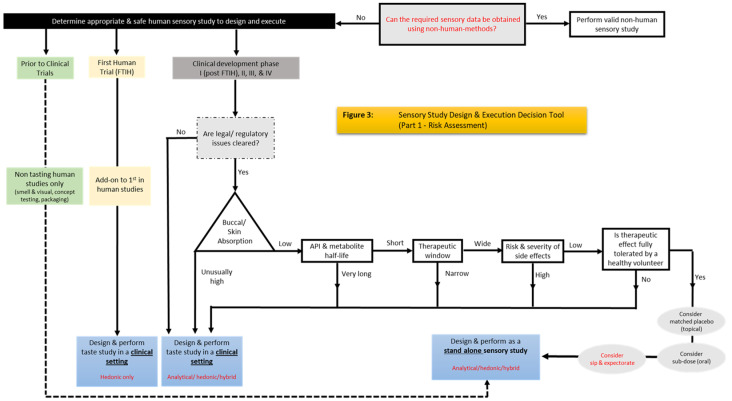
Risk assessment flow chart.

**Figure 4:**
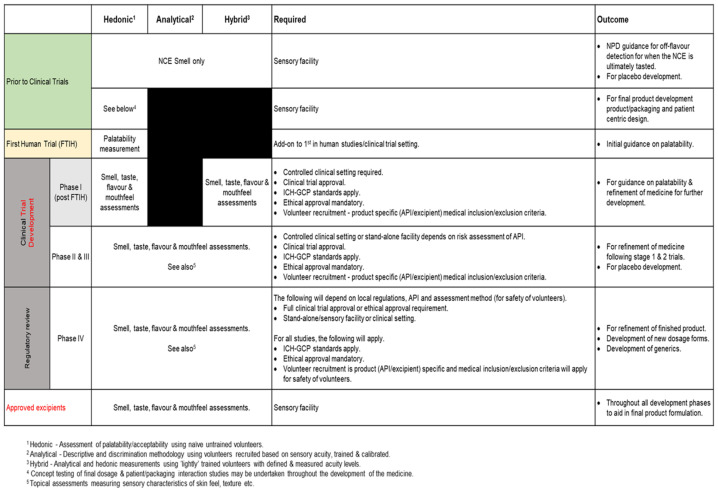
Human Sensory Study Requirements.

Taking the responses from the regulators and our experience in conducting human taste studies during product development into account, we have developed a risk assessment flow chart and a sensory study design and execution decision tool (Figure 3 and Figure 4) to provide a route through the pharmaceutical development process that provides guidance on appropriate and safe human sensory studies.

This risk assessment approach utilises available data concerning the safety and toxicity of the API, its potential absorption, and the risk posed by any small level of absorption that might occur, to evaluate whether the potential study is low risk and can be undertaken in a standalone sensory study, whilst ensuring the safety and wellbeing of the volunteer assessors. Wherever that cannot be assured, the study must be undertaken as part of a clinical trial. The risk assessment procedure must also take into account any specific groups that should not take part in a sensory study, for example, women of childbearing age if reprotoxicity data are not available, or those with a sensitivity to one or more of the formulation components (e.g., penicillin allergy, lactose intolerance, phenylketonurics).

It is also vital that the required sensory information is gained in a scientifically efficient and valid way whilst exposing the volunteer assessor to minimal risk, and that informed consent is obtained. To this end, all proposed studies, whether stand alone or conducted in a clinical setting, will need to be conducted in accordance with a protocol that is approved by the appropriate ethical authorities and will require informed consent from those taking part in the study.

### 4.2. Sensory Study Design and Execution Decision Tool

Any study must use an appropriate sensory study design in order that scientifically valid data are obtained to answer the sensory question being asked. If this is not the case, the formulator could be seriously misled, leading to poor product development decisions and wasting the efforts of the volunteer assessors. This section provides guidance on how to design and conduct sensory studies at different stages of product development.

#### 4.2.1. Introduction

Figure 3 outlines a step-wise risk assessment process that will help determine in which environment the studies can take place (e.g., when a clinical setting is required or when a study can be carried out as a standalone study), as well as, broadly, which method(s) can be used (hedonic (liking/disliking), analytical (detailed sensory studies using trained assessors), or hybrid (sensory studies using experienced assessors); see ‘Panel Selection’ below In Figure 4, the tool is further expanded to outline what can be measured, what is required to carry out the various sensory methods (volunteers/panels, sensory facility/clinical setting, approvals), and the questions these tests/measurements will answer.

The selection of the most appropriate method, use of the correct panel, and controlled testing facilities are basic requirements for sensory analysis studies, regardless of where they are conducted.

#### 4.2.2. What Studies Can Be Undertaken at Each Stage of Product Development?

Analysis of the risks associated with each phase of drug development place limitations as to what and how sensory studies can be carried out. Throughout the phases of drug development and as illustrated in Figure 3 (Sensory Design and Execution Decision Tool Part 2—Human Sensory Study Requirements), we have outlined the sensory methods that are appropriate to use, what is measured using these methods, the requirements to carry out these studies to mitigate risk, and the sensory data outcomes that can be achieved.

- Prior to Clinical Trials

It may be thought that no human sensory data can be obtained prior to the first time in human (FTIH) clinical trial. However, determination of the appearance and key descriptive odour active volatiles through smell assessments of the headspace above an NCE may be undertaken without risk to the volunteer assessor, provided that steps are taken to ensure that no particles can be inhaled. If feasible, such studies can provide useful information to indicate the presence of potential “off-flavours” when the medicine is ultimately tasted. Odour and flavour masking strategies may then need to be considered during formulation development to reduce their impact during early clinical assessment and ultimately make a more palatable product. At this stage, it is typically only possible to provide limited and subjective evaluation of potential palatability. However, further qualitative and quantitively descriptive studies of smell characteristics using individuals with a defined minimum level of sensory acuity and trained to recognise and quantify aroma characteristics of the NCEs in future medicinal development may be possible, if the safety of the API allows.

From this very early stage of development onwards, and further throughout the development process, concept testing of final dosage forms and patient/packaging interaction studies may take place with the target end users (i.e., the patient group and/or their care givers) to inform the Target Product Profile and hence the required sensory attributes of the product.

Where particular patient requirements exist, concept testing allows companies to identify what they are and if these specific requirements are met by the proposed product. This is particularly important for patient-centric drug development, where flexible dosage forms, and routes of administration, are key considerations that may ultimately impact patient acceptability.

- First Time in Human

During the FTIH phase, simple formulations may be used that may inherently limit access of the API to the taste buds (e.g., drug in capsule formulations) which mean that the product is not tasted. However, for some dose delivery routes, such as inhaled products and simple solutions/suspensions, the API will inevitably be present in the mouth and so could be tasted. Any human sensory study undertaken must be carried out as part of the FTIH clinical trial to ensure volunteer safety. Such evaluations can provide basic initial palatability information. Given the small number of subjects involved in such studies, only a limited amount of sensory data can be gathered. Nonetheless, it is important that a properly designed, systematic, and not merely anecdotal, sensory assessment is conducted to provide valid sensory data to guide formulation development for subsequent studies. Unfortunately, it is still not common practice to record sensory information at this stage of development, which is a great missed opportunity. Since the final dose is unlikely to be fully confirmed at this stage, where such studies use a dose escalating design, it would be valuable, where possible, to generate sensory data at each dose since this will help define the taste threshold, and the potential extent of any sensory challenge posed by the API.

It is generally not recognised that poor organoleptic properties, in particular, bitterness, could affect the outcome of these early studies. For example, significant bitterness is related to emesis and drug clearance (potentially leading to poorer drug exposure) and release of stress hormones such as cortisol which could influence clinical observations [48,49,50,51,52,53,54,55,56,57,58,59,60,61,62].

- Product Development Phase—Post First in Human/Phase I

Generally, Phase I sensory research studies focus on palatability aspects of the medicine. Depending on the level of available safety information, they are often included as part of clinical trials, though standalone sensory studies may also be possible.

At this stage in the medicinal product development process, such sensory studies provide valuable research information for further development of the medicine, allowing for consideration of masking strategies, if needed.

Hedonic studies (liking/disliking) or hybrid studies that combine analytical and subjective methodologies to determine both the sensory profile of the medicine as well as its palatability information may be used.

Because of the higher resource levels required (e.g., number of volunteers, screening for sensory acuity, training burden), true analytical studies are rarely used at this stage.

- Product Development—Phase II and III

The majority of sensory studies will be conducted at this stage of the product development life cycle. Objectives for sensory research at the Phase II and III trial stage are often to assess the palatability of the formulation; select flavours and/or sweetener types and levels to aid in improving the characteristics that negatively impact patients’ experience; evaluate taste masking options; evaluate formulation prototypes; or the development of placebos for use in future clinical trials.

At this stage, more safety data are available, allowing more use of non-clinical trial settings, particularly if the “sip and spit” method of tasting is used. While these tasting methods are vital to ensure volunteer/volunteer assessor safety, it should not be forgotten that the sensory experience of the end user of the medicine, i.e., the patient, should always be considered when using such tasting methods. Therefore, the use of proper protocols and volunteer assessor training is imperative to ensure that as many of the sensory characteristics of the medicine as possible are experienced.

For product development studies, the majority of volunteer assessors are likely to be product development scientists, although such studies may also be outsourced to specialist sensory analysis companies. If the outsourcing route is chosen, it remains the responsibility of the pharmaceutical company to ensure that any sensory studies can be carried out safely, for example, by providing sufficient safety data to allow the volunteers to give informed consent (see below).

The studies performed by the assessors can either be truly analytical if suitable qualified volunteer assessors are available (see ’Panel Selection’ below for definitions of volunteer assessors) or, more likely, will be hybrid studies conducted by experienced volunteer assessors. Some companies use inexperienced assessors but provide some (limited) training before the study. The objective will generally be to choose the taste masking methodology (if required), sweetener, and flavour types and levels needed to render the formulation sufficiently palatable to be non-aversive for the intended target population. Quantitative information on all organoleptic aspects of candidate formulations can be gained. Once suitable candidate formulations have been devised, hedonic assessments using naïve volunteer assessors may be required to confirm which, if any, are suitable for use in general clinical trials and/or for the marketed product. This is often necessary since the volunteer assessors may be biased by prior knowledge of the product, particularly if they are the product developers. For example, the candidate formulations they provide for assessment by the naïve volunteer assessors may be greatly improved from the starting formulations, but still not sufficiently acceptable.

Sensory panels may be needed at further points in the development timeline to confirm that the formulation remains acceptable throughout its shelf life. Non-sensory analytical or non-human methods (Section 1 and Section 2) should be used to reduce (or possibly eliminate) the number of these later studies.

The final confirmation that the candidate formulation is acceptable should be assessed by including a sensory evaluation in a clinical trial where the product will be used as intended. This is even more important if the product is intended for a specific patient group, such as paediatric or geriatric patients, since extrapolation from healthy adult panels to these groups may not be straightforward.

- Product Development Phase—Phase IV

Post marketing authorisation sensory studies are often undertaken to aid in the refinement of the finished product, development of new dosage forms (e.g., formulations for different patient groups, modified release formulations, over the counter (OTC) formulations, new flavour variants, and line extensions) or new routes of administration, and generic drug development.

While hedonic, analytical, and hybrid methods are all used at this stage, as with the preceding development phases, whether the research is considered to be a clinical trial or not will depend on the safety profile of the drug, the tasting method used, and local regulations. Given that at this stage of development a considerable amount of information on use of the product in a wide range of human patients is likely to be available, there is an even higher level of assurance that appropriate, standalone sensory studies can be undertaken.

Sensory studies of considerable use during this phase of development include those that compare patients’ assessment of the palatability of existing treatments with the improved version. When the developer produces what they believe to be a more palatable product, this may be “proven” using hedonic assessments.

Moreover, the use of analytical sensory methods that profile sensory characteristic differences can help a developer to choose from a number of options, for example, when masking has reduced bitterness or produced a less gritty product, which of the formulations has the best overall sensory profile.

These studies can also be used to compare the sensory characteristics of the product with other similar products available in the market to either confirm parity/superiority of palatability or to identify areas where improvements can be made.

Whilst generics need to be bioequivalent to the existing innovator product, they do not always have the same sensory profile, either due to the manufacturing route of the API or the excipients used. Hybrid panels may have the dual role of both profiling differences and expressing subjectively which is more palatable. As discussed in our previous paper [9], we strongly suggest that generics developers ensure that their products are at least not organoleptically inferior to the innovator product.

- Excipients

Excipients are major components of medicines and, throughout the process of formulation development, choices are made regarding which excipients to use and at what level. Along with all the other factors, such as safety and technical effectiveness, that need to be taken into account [63], consideration should also be given to understanding excipient sensory characteristics that may ultimately impact the palatability of the final formulation. Special care needs to be taken if a change in an excipient is proposed, even if it is nominally the same material, but from a different supplier, that the organoleptic characteristics of the product remain acceptable.

Should they wish to do so, manufactures of excipients can understand the sensory characteristics of their products by measuring flavour/taste and texture/mouthfeel characteristics using trained panels and sensory descriptive profiling methods. Hedonic measurement of “acceptability” may also be undertaken. Given that the majority of excipients are not pharmacologically active, a wider range of studies are available with fewer constraints due to potential safety issues.

Given the vital importance of excipients and their potential impact on the overall palatability/acceptability of the final pharmaceutical product, there may be a case for pharmaceutical and excipient manufacturers to include meaningful sensory aspects in the specifications for the excipients.

#### 4.2.3. How to Conduct Sensory Studies

- Method Selection

One vital aspect of planning a sensory study is to ensure that the correct test is chosen to address the question being asked. Failure to do so is ethically unsound and may result in erroneous conclusions from the data obtained, which may hinder product development.

The appropriate and safe human sensory studies will in turn depend on (1) the question that needs to be addressed; (2) the phase of drug development; (3) the limitations in terms of panel/volunteer recruitment, facilities required, and panel/volunteer safety; and (4) the level of statistical certainty required in the result.

Some of the most common questions that need to be addressed in pharmaceutical product development and the methods that can be used to address them are summarised in Table 2.

For all tests, it is often possible to obtain additional useful information by including a free-text comment section.

The International Standards Organisation (ISO) (www.iso.org, accessed on 8 September 2023 (ASTM International, formerly known as American Society for Testing and Materials (www.astm.org, accessed on 8 September 2023), have produced a series of standards that describe in more detail the various sensory methods that are generally used throughout the food and cosmetic industries. Those of most relevance to the evaluation of pharmaceutical products are listed in Table 3.

- Panel Selection

Once the required test has been selected, it is then necessary to decide on and recruit the correct number and type of volunteer assessors to address the research objective. Different tests will need different numbers of volunteers. While the ISO/ASTM standards often include guidance on the number of assessments required to provide a statistically valid result, this will not always be either practical or advisable when testing medicinal products. This may be due to stringent safety requirements involved in the recruitment of volunteers, or indeed volunteer availability, suitability, and willingness to participate. To further reduce any risk, as a general rule, the least number of volunteer assessors consistent with addressing the research question should be used. This aspect is discussed further in Appendix B.

Hedonic testing aims to obtain overarching information about products (e.g., how much the product is “liked” or how “palatable” the product is; how does the patient/caregiver interact with the product or its package). For the results to be generally applicable, the panellists are often naïve to the product, having little or no training.

The aim of “Analytical” testing is to obtain objective (often quantitative) data on specific aspects of the sensory attributes of products using panels of trained volunteers. Recruitment and training of such panels can be an extensive process and multiple standards (ISO and ASTM; see Table 3), as well as a large array of textbooks, are available for guidance (e.g., [64]). Different levels of training may be appropriate for different types of analysis of pharmaceutical products. Some individuals will gain experience of what is required by taking part in several sensory studies but have no formal training. These are called “experienced volunteer assessors.”. For more formal sensory studies, some individual panellists will be given formal training in sensory methodologies, vocabularies, etc. These are called “qualified volunteer assessors.”. Often, the qualified volunteer assessors are selected based on heightened sensory acuity. These are called “expert qualified volunteer assessors”. The higher the level of training of the panellists, the fewer that are required. However, a greater training overhead is required to maintain the panel.

For the food industry, the two types of sensory tests are generally regarded as completely separate. Using a descriptive panel to give a hedonic opinion on the product being tested or using a consumer panel to quantify a particular characteristic are rarely, if ever, undertaken.

However, given the small numbers of volunteer assessors necessarily involved in the evaluation of pharmaceutical products, we propose that a third type of study, i.e., hybrid assessments, may also have significant value in this sector. This will allow small panels of experienced (or more highly trained) volunteer assessors to provide quantitative data which, whilst not strictly statistically robust, can still be valuable in guiding product development or to screen candidate formulations to determine which (if any) are sufficiently acceptable/palatable to justify further testing in larger panels, regardless of whether these are hedonic or analytical. Statistically robust studies may be required when an adult panel screens candidate formulations before deciding which (if any) can proceed to testing in paediatric patients/volunteers as part of a paediatric clinical study (with palatability evaluation as a part of it).

When taste, flavour, or mouthfeel characteristics of products are being assessed and ingestion or sip and spit studies of the products are required, not only do the panellist/volunteers need to be recruited based on their sensory acuity, but any medical inclusion and exclusion criteria also need to be satisfied. In addition, it will be necessary to obtain informed consent from each volunteer based on an understanding of the toxicology data that is available for the API and the burden to participants, for studies performed as standalone sensory studies, as well as those performed as part of a clinical trial.

#### 4.2.4. Documentation and Performance of the Study

In order to be scientifically valid and ensure the safety of the volunteer assessors, the study will need to be performed in conformity with a suitable protocol. If the study is performed as part of a clinical trial, then appropriate aspects from those outlined below should be included in the clinical protocol.

If the study is a standalone sensory study, then ideally all steps required to actually undertake any sensory study should be covered by a Standard Operating Procedure (SOP), which should include relevant Good Clinical Practice (GCP), Good Manufacturing Practice (GMP), and General Data Protection Regulations (GDPR) aspects.

All in-house standalone sensory studies MUST be conducted in accordance with a written and approved protocol which should include the following elements. If the study is performed at a third party, such as a sensory analysis consultant/company, the commissioning pharma company must assure themselves that the way that the third party intends to conduct the study and the controls that they intend to use are sufficient to address these elements.

- The Study Aims and Objectives

Which must be clearly articulated

- The Number and Type of Volunteer Assessors

Which must be justified and include, where appropriate, inclusion and exclusion criteria.

- Informed Consent Procedure

This must include the information that will be provided to volunteer assessors in order to enable them to provide that informed consent. The information must be provided in a format that is fully intelligible to any potential volunteer. For example, if the potential volunteers are pharmaceutical development scientists, they may reasonably be expected to understand technical terms that may be used in toxicological data summaries, etc. However, if this is not the case, the information should be presented in “layman’s language”. For any studies conducted, it is vital that participation is entirely voluntary, with no overt or covert pressure to take part. Informed consent information will also contain information concerning how much time will be required, any constraints upon volunteer behaviour (e.g., the need to refrain from eating certain foods or not smoking), any foreseeable adverse effects of taking part, and what to do in the unlikely event of any adverse effect of the product.

- How Samples will Be Prepared and Handled

The preparation of the test samples of each product studied must be undertaken by competent staff in accordance with a batch record that is overseen by Quality Assurance. This might either be as part of a normal manufacturing campaign or as part of “chair side”/”extemporaneous” manufacture. If chair side manufacture is used, the preparation area must be clean and clear of all other products. The extemporaneous preparation must be completed under strict quality measures in a dedicated laboratory according to approved SOPs, and, where necessary, be supported by stability data covering the production and testing period.

All people involved in sample preparation must be qualified and properly trained. Every manufacturing step of the batch document (weighing, addition of liquid, mixing, etc.) must be documented, performed by one individual, and witnessed by another to ensure that mistakes are not made. All materials used are released for human use, clearly packed, labelled, and stored in a proper manner, avoiding any contamination, and in storage conditions supported by stability data. Prepared samples must also be clearly packed, labelled, and stored in a manner that ensures they cannot be contaminated nor confused with each other. Samples will need to be QA released and assigned a shelf life based on available data. If the samples are prepared as part of a standard manufacturing campaign, the shelf life will be that of the batch. For chair side manufacture, the shelf life will be 24 h unless available data supports a longer shelf life.

Each sample must be prepared to be distributed individually to each panellist and identified by a random code with at least three digits. Each sample must be presented in such a way that each panellist has access to the correct amount of sample to be studied without possible error (e.g., 5 mL of a liquid, the correct number of tablets, the amount of granule required, etc.). One way of achieving this would be to present the volunteer with a “unit dose” sample.

It is also necessary, if several samples are to be tested in the same session, that other products are available to the panellists to rinse the mouth and neutralise any other tastes that may be present (for example water, azyme bread, or dry crackers).

The protocol should also contain instruction of how any unused material should be safely disposed of.

- Facilities where the Test Will Take Place

The human response to one sensory stimulus cannot easily be separated from that to other stimuli present at the same time.

Thus, the physical environment where the test will take place should be designed to minimise any distractions that may interfere with the assessments being undertaken. Therefore, there should not be excessive noise, it should be neither too hot nor too cold, and the volunteer assessors should not be exposed to extraneous aromas, etc. The space should be designed so that each individual volunteer assessor cannot be influenced in their assessment by any other volunteer assessor. This can be achieved either by volunteer assessors entering the test space one at a time or using screening.

It is also important to consider how to control for all other aspects of the product that the volunteer assessor will be exposed to when asking them to comment on a particular sensory aspect. For example, if the samples differ in colour as well as flavour this could bias the results. The use of coloured lighting could mask these differences in appearance. Other options are to use opaque or coloured packaging such as amber bottles and syringes.

On the other hand, if the assessment is a hedonic assessment of the overall acceptability, it might be appropriate to accept any differences in appearance between the test articles.

- Instructions to the Volunteer Assessors

These will translate the steps required to undertake the chosen sensory study type into step-by-step instructions for the volunteers. For example, these instructions will include how the volunteer should prepare their palate prior to taking the sample, what quantity (volume/number of dose units) of sample they should use, how long they should keep it in their mouth, where they should spit it out, how to record their assessment, how to cleanse their palate before assessing subsequent samples (if any), and how long to wait between samples. If the test is for a topical product, the instructions should define the area of skin to be used, how to apply the product, how long to leave it in contact with the skin, and how to clean the skin after the assessment.

Regardless of whether the sample is assessed orally or topically, the instructions should be designed to ensure minimal systemic exposure to the product (unless toxicological data permits otherwise). For an oral product this will usually require a “rinse and spit” design with a short residence time in the mouth. For a topical product, the area of skin exposure should be small and the exposure time should be short.

Where more than one sample is being assessed, the order in which they are to be assessed must be randomised and specified. Care must be taken to balance the presentation order so that each sample is assessed in a particular order (e.g., first, second, or last) an equal number of times. It is also important that the samples are identified by a random code in order not to imply a preference order to the volunteer assessor.

Instructions to the volunteer assessor MUST also include action to be taken in the event of accidental ingestion of the product or a suspected adverse reaction.

If the study is well designed, the risk of any adverse event is extremely small. Nonetheless, it is wise to have trained first aiders/medical oversight readily available as an added precaution.

- How the Data Will Be Captured

Written comments permit the sensory sensations to be described whilst measurement scales allow them to be quantified. Free-form comments from volunteer assessors are valuable in addition to the quantitative data. However, care must be taken to ensure that the person interpreting the vocabulary used fully understands what the volunteer assessor was trying to convey. There are no published standards for the vocabulary to be used when assessing pharmaceutical products. It is however possible to agree certain terms with an experienced or trained panel, whilst comments made by untrained volunteer assessors can be clarified with follow-up questions if required. The most common confusion of terms in the evaluation of medicinal products is between actual bitterness and other similar sensations such as sourness and astringency. It is therefore important to select a vocabulary that is understood by the panellists and that allows an optimal characterisation of the organoleptic aspects of the product.

The intensity of perception can be captured using various scales including visual analogue scales (a so-called continuous scale) and category scales such as tick boxes. There is a considerable literature on the subject of the best methods to capture data from these studies, which is outside the scope of this paper. Interested readers are referred to these papers for more information [65,66]. Fundamentally any appropriate scale can be used provided that the volunteer assessors understand it and have confidence in using it.

Hedonic scales are well tried and tested for capturing palatability/acceptability data [67]. The typical example is the nine-point hedonic scale, a version regularly used with consumers in preference mapping studies to capture “liking” scores. The hedonic scales are also appropriate for testing children 5–12 years of age, often using a 3-, 4-, 5-, or 7-point “frowning to smiley” face scale [68,69]. Older children can also use VAS scales [70]. For younger children, it may be necessary to use trained observers or automated facial recognition methodologies to assess the child’s reaction from their facial responses and their action (e.g., whether they grimace or spit out the sample) [71,72]. Where the sample can actually be taken by a child, the CAST system [73], which has recently received a letter of support from the EMA for acceptability testing [74], can provide a structured, multifaceted assessment of overall sample acceptability [75,75,76,77,78,79,80,81,82,83,84]. Implementing an acceptability testing strategy as soon as possible in product development is important to better understand the many aspects—not limited to sensory aspects—of this multi-faceted concept.

- How the Data Will Be Analysed

In our survey of regulators (Appendix A), none of the agencies have any guidance on what data derived from human sensory evaluation studies should be provided and/or the format in which the data should be presented (Q5). Each instance is considered on a case-by-case basis. This is equally true whether the data are for the product alone or claiming superiority over another product (Q7). Interestingly, none of the agencies have any guidance on the overall proportion of respondents that must find the product to be palatable for the palatability to be considered acceptable for product approval (Q10).

Traditionally, products of the food, beverage, and fast-moving consumer good (FMCG) industries are pleasant. Pharmaceutical products typically are not and arguably should not be. Rather, they should be non-aversive. This should be taken into account when analysing the data.

Descriptive data should be summarised and any trends in the comments identified and reported.

Numerical data should be summarised. For any data set, it should be possible to calculate the mean and standard deviation for each sample or descriptor assessed. Where possible, the data should also be statistically analysed. At the simplest level, this will consist of identifying whether the conditions have been satisfied to conclude that a sample is preferred to another/is different to another, etc. Then a normality test may be carried out in order to examine the statistical tests that can be used to compare products. In the case of a standard distribution, multivariate analyses such as ANOVA can be used. In the case of a non-normal distribution, other statistical approaches should be used, such as the Friedman test.

More sophisticated tests can sometimes be used to test whether the data set allows more detailed examination, for example, whether there is a sample order effect (e.g., whether the sample tested first is always preferred or all samples tasted after a particular one are always considered worse), or if there is a difference between the responses provided by different volunteer assessor groups (e.g., male/female, smoker/non-smoker).

For the CAST methodology of assessing medicine acceptability, the data are presented graphically on the acceptability reference framework which includes several real-life “observer-reported outcomes” collected for many medicine intakes in paediatric subjects. “Outcome measures”, such as whether the product was actually taken with or without any further incentive, whether it was spat out, or whether it was totally rejected, are recorded. Each evaluation combines several observed behaviours (e.g., required dose intake, time needed, patient reaction, use of food/drink to mask a bitter taste, crushing a tablet which cannot be swallowed) to reflect the ability and willingness of patients and caregivers to use (preparing and administering) medicines as intended.

- Data Reporting

Sensory analysis study reports should include all the elements of the studies predefined in the protocol: the methods, the study locations and dates, the panels and their objective, the questions, and the results obtained. These should be written in a manner that is readily interpreted by a trained scientist but who is not a sensory analysis expert, using graphical methods to illustrate results and conclusions, e.g., histograms, spider graphs, or mappings.

All documentation, including the identity of the individual volunteer assessors, should be archived according to the policy of the institution undertaking the study and in compliance with applicable data protection requirements.

#### 4.2.5. Paediatric Specific Aspects

The majority of product development studies will be performed using adult volunteers for ethical and practical scientific reasons.

Nevertheless, data from adults alone may not be sufficient to ensure that a product will have acceptable sensory characteristics for a child. Whilst regulators will sometimes accept data in adults as supporting data concerning product acceptability (Q6—regulator survey; see Appendix A and Supplementary Materials data, File S1), it will almost always be necessary to back this up with data in the target population, i.e., children of the appropriate age range. Such studies will normally be hedonic studies conducted in a clinical setting and using simplified methods of gathering the sensory data.

Due to their potential increased level of vulnerability to the effects of any API that is absorbed during sensory studies, enhanced ethical scrutiny may be required for studies performed in children. Factors that may need to be considered are the following:

- The age/developmental status of the child. ADME (absorption, distribution, metabolism, and excretion) aspects change with age. Young children may be more vulnerable to any adverse effects from any API that is absorbed. Older children are more likely to be similar to adults in this regard. A thorough knowledge of the toxicological effects (including, where possible, developmental effects) of the API/formulation is required in order to evaluate the nature/magnitude of any increased risk. For this reason, most early studies are undertaken in adults. Unless this evaluation clearly demonstrates that the study is low risk in children, it will also need to be undertaken under full clinical supervision.
- The capability of the child—this will influence whether they can be expected to understand and follow the study instructions (influencing how samples should be given) and how to record their responses. Different methods of gathering responses to ensure that scientifically valid data are generated are appropriate at different stages of development (often, but not always, linked with age).
- Informed consent—ideally this will be provided by the child themselves, although it may be necessary for this to be sought from the parent/carer. The topic of informed consent in this demographic is a large one. A detailed discussion is outside the scope of this paper.

Nevertheless, the same principles as described above can be applied to ensure the scientific integrity of any sensory studies performed in children. Some interesting papers on conducting sensory research with children are available [85,86], although these deal with food products, and hence do not have the same level of toxicological issues that the evaluation of medicines will often entail. The issue of how best to capture the data from sensory studies in children is an ongoing debate. This is briefly discussed above. An additional helpful review [87] and best practice recommendations [88] are available for the interested reader.

## 5. Discussion/Conclusions/Recommendations

In this paper, we propose standards and methodologies to undertake sensory studies of pharmaceutical products. There is a development need for an internationally harmonised methodology, guidance, standardisation, and tailored documentation for all stages of new medicinal product development, specific to the pharmaceutical industry, which adapt and combine traditional sensory methods to meet the needs for developing medicinal products. We hope that this paper provides a useful stimulus for the development of those standards. The main advantage of conducting studies according to agreed standards would be to allow data to be compared from studies conducted in different laboratories, simplifying the task of regulators in assessing the acceptability of products and allowing different pharmaceutical manufacturers to drive their search for optimal formulations.

To aid readers to see how the principles outlined in this paper can be used in practice, the authors intend to provide some worked examples of their application in a follow-on paper.

## Figures and Tables

**Table 1:** Comparison of the benefits and/or advantages and the challenges of the three study types.

Factor	Study Type		
	Pre-Planned Clinical Trial with Additional Sensory Endpoint	Sensory Specific Clinical Trial	Standalone Sensory Study
API exposure	Full exposure to product/API possible	Full exposure to product/API possible	Design limits exposure
Participant risk	Full clinical monitoring Higher risk studies possible	Full clinical monitoring possible, ethically only low to moderate risk studies possible	Low risk studies only
Participant type/number	Probably naïve/ could be many	May be trained/ very few	Full range of participant type/few
Focus	Sensory data secondary	Sensory data primary	Sensory data primary
Sensory data type	Simple/hedonic only	Simple/hedonic Limited hybrid	Full range
Sample throughput	Low	Low	Moderate to high
Cost	High	High	Moderate to Low
Complexity	High	High	Moderate
Flexibility	Low	Low	High
Frequency	Low	Low /moderate	High
Design ease	Low	Low /moderate	High

COLOUR KEY. Green = benefit and/or advantage, Amber = moderate challenge, Red = high challenge—from a product development/sensory analysis viewpoint.

**Table 2:** Sensory questions in pharma development and methods to address them *.

Question	Possible Methods	Comments
What is the sensory acceptability of the product as a whole?	Acceptance tests, paired comparison, or ranking tests.	A range of methods of capturing data are available.
What is the acceptability of some specific aspects of the product?	For specific aspect assessments use bimodal visual analogue scales (VASs) **	Data captured by volunteer marking a scale that has “too little” at one end of the scale, “too much” at the other, and “just right” in the middle.
Is one product better than another?	Paired preference test (two samples) Or Ranking test (more than two samples)	When the difference between products is expected to vary according to only one attribute (e.g., bitterness), orientated questions such as “Which sample is the most bitter?” may be used.
What level of a specific characteristic(s) are perceived?	Profiling methods or Rating scales	Continuous methods such unimodal VAS scales (e.g., when measuring the level of bitter taste, the assessor marks on a scale their perception of the level of the bitter taste from none to extreme, or low to high, etc.). Categorical methods e.g., assessor ticks predefined boxes.
Is one sample different to another?	Triangle or Duo Trio test	Most useful when comparing samples that are nominally the same, e.g., samples from two batches of the same product, or new and aged samples of the same product. These sensory methodologies avoid volunteer assessor bias by simply asking if the samples are different.

* The sensory data may be combined with data concerning other acceptability aspects of the formulation to better understand the many aspects of this multi-faceted concept. ** VAS = visual analogue scale.

**Table 3:** Sensory analysis standards.

ISO Standard Number ^1,2^	Standard Name and Description
ISO 6658:2017	Sensory analysis—Methodology General guidance on the use of sensory analysis. It describes tests for the examination of foods and other products by sensory analysis and includes some general information on the techniques to be used if statistical analysis of the results is required.
ISO 8589:2007	Sensory analysis Provides general guidance for the design of test rooms intended for the sensory analysis of products.
ISO 13300-1:2006	Sensory analysis General guidance for the staff of a sensory evaluation laboratory—Part 1: Staff responsibilities.
ISO 13300-2:2006	Sensory analysis General guidance for the staff of a sensory evaluation laboratory—Part 2: Recruitment and training of panel leaders.
ISO 11136:2014	Sensory analysis—Methodology General guidance for conducting hedonic tests with consumers in a controlled area.
ISO 13299:2016	Sensory analysis—Methodology General guidance for establishing a sensory profile.
ISO 5492:2008	Sensory analysis—Vocabulary Defines terms relating to sensory analysis. Applies to all industries concerned with the evaluation of products by the sense organs. The terms are given under the following headings: (1) general terminology; (2) terminology relating to the senses; (3) terminology relating to organoleptic attributes; and (4) terminology relating to methods.
ISO 4121:2003	Sensory analysis Guidelines for the use of quantitative response scales. Provides guidelines describing quantitative response scales (where the response obtained indicates the intensity of perception) and their use when assessing samples.
ISO 8586:2012	Sensory analysis General guidelines for the selection, training and monitoring of selected and expert volunteer assessors.
ISO 11132:2021	Sensory analysis—Methodology Guidelines for the measurement of the performance of a quantitative descriptive sensory panel.
ISO 3972:2011	Sensory analysis—Methodology Method of investigating sensitivity of taste.
ISO 5496:2006	Sensory analysis—Methodology Initiation and training of volunteer assessors in the detection and recognition of odours. Describes several types of method for determining the aptitude of volunteer assessors and for training volunteer assessors to identify and describe odoriferous products.
ISO 4120:2021	Sensory analysis—Methodology Triangle test. Specifies a procedure for determining whether a perceptible sensory difference or similarity exists between samples of two products.
ISO 13301:2018	Sensory analysis—Methodology General guidance for measuring odour, flavour and taste detection thresholds by a three-alternative forced-choice (3-AFC) procedure.
ISO 8588:2017	Sensory analysis—Methodology “A”—“not A” test. Specifies a procedure for determining whether a perceptible sensory difference exists between samples of two products. The method applies whether a difference exists in a single sensory attribute or in several.
ISO 10399:2017	Sensory analysis—Methodology Duo-trio test. Specifies a procedure for determining whether a perceptible sensory difference or similarity exists between samples of two products.
ISO 8587:2006	Sensory analysis—Methodology Ranking. Describes a method for sensory evaluation with the aim of placing a series of test samples in rank order.
ISO 5495:2005	Sensory analysis—Methodology Paired comparison test. Describes a procedure for determining whether there exists a perceptible sensory difference or a similarity between samples of two products concerning the intensity of a sensory attribute.

^1^ ISO standard number and year of issue (typically, ISO sensory standards are reviewed regularly (~5 years). When no changes are made the year of issue remains the same). ^2^ ASTM standards are also available www.astm.org accessed on 8 September 2023.

## Data Availability

Please see Supplementary data for the new data generated as part of this study.

## Supplementary Materials

The following supporting information can be downloaded at: https://www.mdpi.com/article/10.3390/pharmaceutics15092319/s1, File S1: Report Regulatory Survey EuPFI Palatability.

## Appendix A. Survey of Regulators—Summary of Responses

A 10-question survey was compiled to seek to understand the requirements of regulators with respect to sensory/palatability studies. The survey was sent to contacts in 32 national regulatory authorities, of which 13 countries replied (a response rate of 41%). Responses were received from all areas of Europe. Attempts were also made to obtain a response from the FDA, but this proved not to be possible.

Detailed results obtained are available in the Supplementary Materials, File S1. A brief summary is provided below:

Q1. *Do you agree that a “swirl and spit” sensory study is not a Clinical Trial of an Investigational Medicinal Product (IMP) as defined by the EU Directive 2001/20/EC?*

As pointed out in several of the responses, guidance in EU Directive 2001/20/EC defines what is regarded as a clinical trial [52]. Based on this guidance, the majority of the respondents (69%) agreed that a “rinse and spit” design study, where drug absorption risk is low, does not fall within the definition of a clinical trial. Some agencies also referred to a decision algorithm e.g., [55] concerning what is and is not regarded as a clinical trial. In particular, one agency clarified that “taste is not considered a safety or efficacy endpoint”. The other respondents indicated they had little experience of such studies and would wish to make the decision on a case-by-case basis.

Some respondents pointed out that if significant exposure to the API is expected, then sponsors should consider gaining sensory data as part of a clinical trial. The risk assessment process we propose below takes this into account.

Q2. *Do such “swirl and spit” studies require an authorization from, or a notification to, your national agency?*

Only a minority of agencies require rinse and spit studies to be notified to the agency, although all would require ethical approval of such a study.

Q3. *Is there another entity or governing body (apart from ethical approval) to whom such studies should be referred to or would manage/control/oversee these studies in your member state?*

The wide variety of responses suggested that this question was not sufficiently clear.

Q4. *If your agency does monitor such studies would your requirements differ if the material being tested is a new chemical entity or an established product?*

The majority of respondents indicated that they did not have sufficient experience of such studies to form a view but that in principle requirements do not differ whether the drug is an established one or a new chemical entity (NCE)

Q5. *Does your national agency have any requirements for what data should be provided and/or the format in which the data should be provided that is derived from human sensory evaluation studies? In addition: Q7 What evidence would your national agency ask for if a sponsor wished to make a claim about the sensory aspects of their product (for example that the product has superior palatability to a previous formulation)?*

None of the agencies have any guidance on what data derived from human sensory evaluation studies should be provided and/or the format in which it should be presented. Each instance is considered on a case-by-case basis. This is equally true whether the data is for the product alone or claiming superiority over another product (Q7).

Q6. *Would your national agency consider data from palatability assessments performed with a panel of healthy adult volunteers to be adequate data to support evidence of palatability in the targeted population (e.g., paediatric) in the drug development dossier?*

Interestingly, the agencies were roughly evenly split on whether data from adult panels would be considered sufficient for establishing acceptability in a paediatric population. However, they reported that this may be accepted with justification either alone or as supportive evidence along with data from the target population. Ideally, all authorities would like to see data from the target population in the drug development dossier.

Q7. See Q5 above.

Q8. *Would a similar approach be taken for other non-oral organoleptic sensory studies, e.g., for topical/transdermal products, nasal/inhaled products?*

Although the intent of this question was to understand whether all sensory studies, whether for taste or sensory characteristics of products administered by non-oral routes, would be viewed the same way by each of the regulators, it is possible that the question may have been linked to Q7 regarding claims of superiority. However, where the detailed response from the agency indicated that they had interpreted the question in the way we had intended, the majority suggested that the same approach would be taken for sensory studies irrespective of the administration route.

Q9. *Some sensory data are available using non-human studies, for example, e-tongue and rodent BATA (brief-access taste aversion) studies.*

*Does your agency accept such data as evidence of the palatability of formulations?* There was unanimity across the responses that non-human sensory data alone (e.g., e-tongue, rat BATA) would not be acceptable as sole evidence of the palatability of a formulation, and 17% of respondents suggested that it would not even be accepted as supporting evidence. Some said they would consider these data in support of a human study if scientifically justified, although most of the agencies said that they had little or no experience of evaluating such data.

Q10. *In any sensory study there will be a proportion of respondents who find the product to be palatable and a proportion that do not. Does your agency have any guidance on the overall proportion of respondents that must find the product to be palatable for it to be approved?*

None of the agencies have any guidance on the overall proportion of respondents that must find the product to be palatable for the palatability to be considered acceptable for product approval.

In summary, it appears that standalone sensory studies are permitted in many jurisdictions, although perhaps not in others, provided that they are assessed as low risk and have ethical approval.

## Appendix B. Number of Volunteer Assessors Required

There are no agreed standards for sensory science as it applies to the pharmaceutical industry and requirements with regard to numbers of volunteers necessary for each method do not exist. The methods and guidelines currently in use for pharmaceutical products are, for the most part, those already in use to assess FMCGs, in particular in the food and beverage sectors. However, the recruitment of the same or similar numbers of volunteers to assess pharmaceutical products is at present unlikely to be a common occurrence [89,90].

Depending on the phase of product development, the safety profile of the API, the method selected, and the type of volunteers needed for a particular method, the pharmaceutical industry generally takes a pragmatic approach to recruitment of volunteers, and therefore this number may differ substantially from international agreed norms for FMCGs (Table 3).

Due to the lack of internationally agreed standards, there remains a significant gap which will need to be addressed to provide the best practice for developing patient-centric, age-appropriate palatable pharmaceutical products.

Table A1 exemplifies the number and type of volunteers required for different sensory analysis studies. It takes into account the pragmatic requirements of pharmaceutical sensory studies. As such, it may differ substantially from the fully statistically valid studies often undertaken in the food and beverage industries.

**Table A1:** Assessor numbers.

The Questions	Method Type	Panel Selection	Minimum of Volunteers Typically Used	Number in the ISO Norms Typically Used for FMCG
What is the acceptability of the whole product or some aspect of the product? Is one product better than another? Using the preference test	**hedonic**	Recruited based on inclusion/exclusion criteria. No training required or experience needed.	Less than 10 for pre-screening of very different formulations. Generally, 30 (for acceptability of the whole product).	100 (for acceptability of the whole product). 60 (for a ranking test) ISO:8587 ** 24–30 (for a paired comparison test) ISO:5495.*
Is one sample different to another?	**Analytical—discrimination test**	Recruited based on inclusion/exclusion criteria. Volunteers require minimum defined levels of sensory acuity. Method training then required.	Less than 10 for pre-screening of very different formulations.	12–15 (for a ranking test) ISO:8587. ** 24–30 (for a paired comparison test) ISO:5495.*
How are products different to each other	**Analytical—descriptive test**	Recruited based on inclusion/exclusion criteria. Volunteers require high levels of sensory acuity. Training then required to profile sensory characteristics of the products.	Less than 5 for pre-screening of very different formulations. Generally, 8–12.	8–15 Quantitative Descriptive Profile (ISO:13299).

* If 10 assessors are available, have each assessor perform three paired tests to obtain a total of 30 evaluations.
