# Supplementary material for: A Guide to Best Practice in Sensory Analysis of Pharmaceutical Formulations
Source: Pharmaceutics. 2023 Sep 14;15(9):2319. doi: 10.3390/pharmaceutics15092319 (PMC10535428; doi:10.3390/pharmaceutics15092319)
Supplement: Supplementary file 1 [file pharmaceutics-15-02319-s001.zip › pharmaceutics-2566351-supplementary.pdf]

File S1

Report Regulatory Survey EuPFI Palatability

Last update
23/11/2022

Countries that have replied

- **13 countries in total**

    Croatia
    Czech Republic
    Estonia
    Germany
    Ireland
    Italy
    Lithuania
    Norway
    Poland
    Romania
    Slovenia
    Sweden
    Switzerland
    United Kingdom

1. Do you agree that this is not a Clinical Trial of an Investigational Medicinal Product (IMP) as defined by the EU Directive 2001/20/EC?

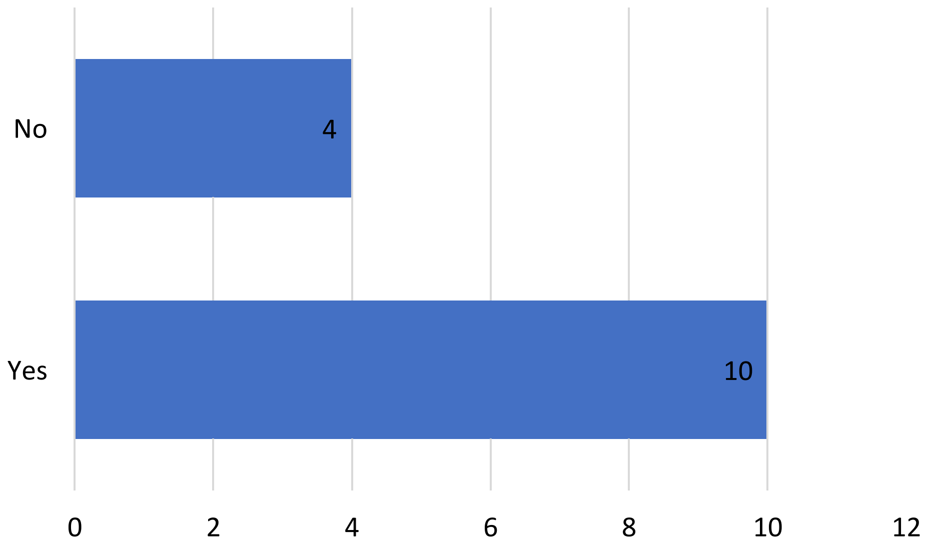

Croatia, Germany, Romania, Lithuania

Czech Republic, Estonia, Ireland, Italy, Norway, Poland, Slovenia, Sweden, Switzerland, UK

Answer	%	Count
Yes	71%	10
No	29%	4
Total	100%	14

Explanation to Q1

Country	Explanation
Croatia	This is the position of Croatian Agency for Medicinal Products and Medical Devices. Please note that the national competent authority for authorisation of clinical trials in Croatia is Ministry of Health.
Czech Republic	N/A
Estonia	This description does not fulfil definition for clinical trial according to the EU Directive 2001/20/EC.
Germany	In Germany this decision has to be filed by the national local authorities the so called Landesbehörden. They are responsible for the final decision if a study is classified as a clinical trial requiring approval according to Directive 2001/20/EC. However according to the definition of the Directive 2001/20/EC we would not classify a swirl and spit study as a clinical trial.
Italy	Conversely, according to CTR 536/2014 Q&A Annex 1 Decision tree to establish whether a study is a "clinical trial", https://ec.europa.eu/health/system/files/2022-05/regulation5362014_qa_en.pdf - Column C encompasses trials on "palatability" intended to assess the suitability of a formulation for a particular population.
Poland	The basic drug is still regulated by NCA, so changing formulation doesn't change the drug itself.
Romania	Generation of the palatability data is included in most of the cases as one of the objective of the clinical trial rather than in a dedicated study.

(continued)

Explanation to Q1

(continued)

Country	Explanation
Slovenia	Not in the scope of definition of a »clinical trial« as laid down in EU Directive 2001/20/EC.
Sweden	In general, we do not consider swirl and spit studies to be a clinical trial on medicinal products for which an approval from the competent authority is required given that the substances do not have time to be absorbed during gargling, but in cases where there is a risk of swallowing the substance or if there is a risk of absorption thorough the mucous membrane the study may be subject to a permit.
UK	Taste is not considered a safety or efficacy endpoint (Efficacy is the concept of demonstrating scientifically whether and to what extent a medicine is capable of diagnosing, preventing or treating a disease.); also, as the product is spat out again it could be argued that the test product does not function as a medicine (i.e. it does not restore/correct/modify physiological functions by exerting a pharmacological, immunological or metabolic action or to making a medical diagnosis or is otherwise administered for a medicinal purpose).
Ireland	The decision tree contained in the guidance documents related to the CT directive mentions efficacy safety and pharmacokinetics, none of which seem to be involved in studies of this type.
Norway	N/A
Lithuania	It will depend on the particular situation and product. EMA guidance states: Any modification of the preparation will change the pharmaceutical characteristics of the preparation as studied in and justified by the clinical trials and (bio)pharmaceutical studies. Therefore it is essential that every modification is verified with respect to its potential impact on the safety and efficacy of the medicinal product (page 8).

Explanation to Q1

(continued)

Country	Explanation
Switzerland	It is palatability studies for new flavoured medicines, particularly when a swirl and spit method is employed

2. Do such ‘swirl and spit’ studies require an authorization from, or a notification to, your national agency?

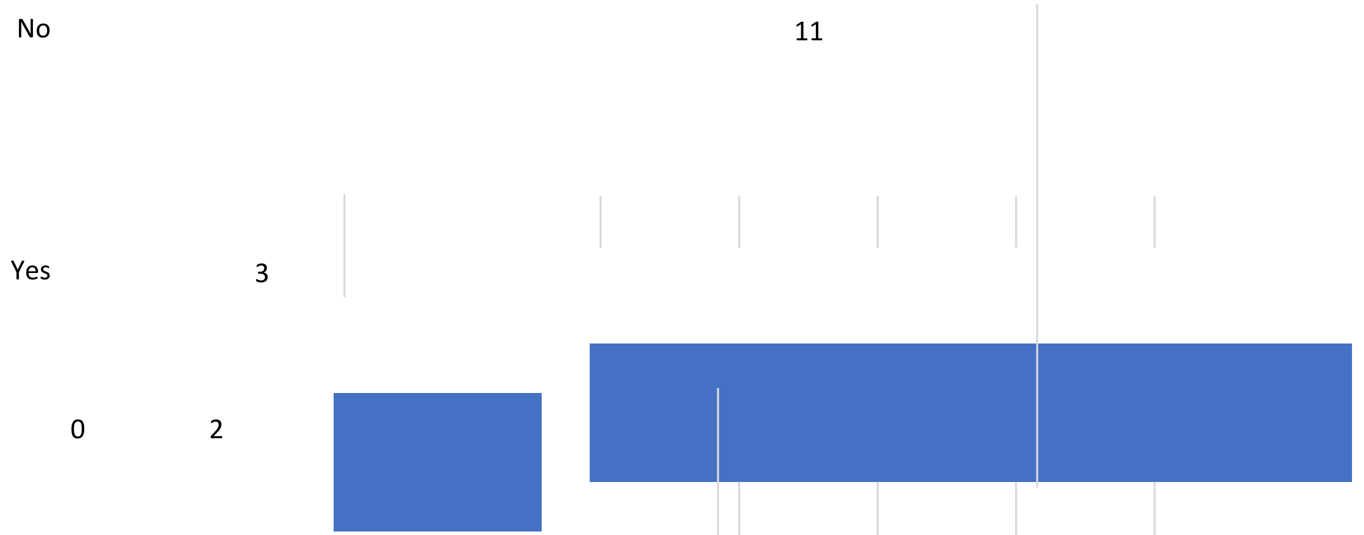

Croatia, Czech Republic, Estonia, Germany, Italy, Norway, Slovenia, Sweden, UK, Lithuania, Switzerland

Poland, Romania, Ireland

Answer	%	Count
Yes	21%	3
No	79%	11
Total	100%	14

Explanations to Q2

Country	Explanation
Croatia	Each clinical trial needs to be authorised by competent authority. Please note that the national competent authority for authorisation of clinical trials in Croatia is Ministry of Health.
Czech Republic	N/A
Estonia	N/A
Germany	In Germany this decision has to be filed by the national local authorities the so called Landesbehörden. They are responsible for the final decision if a study is classified as a clinical trial requiring approval according to Directive 2001/20/EC. However according to the definition of the Directive 2001/20/EC we would not classify a swirl and spit study as a clinical trial.
Italy	Currently according to Directive 2001/20/EC not required.
Poland	Yes because its still study on drug.
Romania	To my knowledge such studies follow a protocol established in advance that requires an authorization from the national agency.
Slovenia	We haven't had any notification so far. If these kinds of studies are defined in EU/national legislation then an authorization/notification would be necessary.
Sweden	N/A
UK	As these kind of swirl and spit studies are not considered to be clinical trials under The Medicines for Human Use (Clinical Trials) Regulations 2004 as amended, they do not require a CTA.

(continued)

Explanations to Q2

(continued)

Country	Explanation
Ireland	As it is not a clinical trial, it does not come within the remit of the NCA.
Norway	N/A
Lithuania	Yes - If it is a part of clinical trial.
Switzerland	We have not got and done such intervention and not mentioned in our guidelines for clinical trials reviews

3. Is there another entity or governing body (apart from ethical approval) to whom such studies should be referred to or would manage/control/oversee these studies in your member state?

Country	Answer
Croatia	Each clinical trial needs to be authorised by competent authority. Please note that the national competent authority for authorisation of clinical trials in Croatia is Ministry of Health.
Czech Republic	we are not aware of such other entity (apart from ethical approval).
Estonia	Estonian Agriculture and Food Board oversees topics related to food and food supplements.
Germany	The local authorities "Landesbehörden" may be involved in the classification of a study as clinical trial. If a study is classified as a clinical trial they are also responsible for the control of this clinical trial.
Italy	No.
Poland	Only NCA.
Romania	Approval from the national agency is required.
Slovenia	Ethics Committee.
Sweden	No.
UK	HRA approval should be sought if the project is considered to be research. The HRA is the governmental body for ethical approval of research in humans. MHRA is not involved in this area of regulation and so cannot comment on whether a palatability study would require approval from HRA or other governing bodies.

(continued)

3. Is there another entity or governing body (apart from ethical approval) to whom such studies should be referred to or would manage/control/oversee these studies in your member state?

(continued)

Country	Answer
Ireland	Not that I am aware of.
Norway	No, not that I am aware.
Lithuania	No.
Switzerland	Not to my knowledge.

4. If your agency does monitor such studies would your requirements differ if the material being tested is a new chemical entity or an established product?

Country	Answer
Croatia	Each clinical trial needs to be authorised by competent authority. Please note that the national competent authority for authorisation of clinical trials in Croatia is Ministry of Health.
Czech Republic	In the case of testing an unauthorised product, this would be assessed as a part of clinical trial. In the case of flavour testing an authorised product, we do not assess these studies.
Estonia	N/A
Germany	N/A
Italy	Palatability studies have not been submitted to our Agency so far, however, it is agreeable that the requirements would differ if the material being tested is a new chemical entity or an established product.
Poland	YES, it's obligatory.
Romania	No, the requirements are the same no matter if a new chemical entity or an established product is tested.
Slovenia	Our Agency doesn't monitor these studies currently. If compared to clinical trials the requirements for a new chemical entity would be broader.
Sweden	The requirements will be the same irrespectively if it is a new chemical entity or an established substance.
UK	N.A. as study is not monitored by MHRA.

(continued)

4. If your agency does monitor such studies would your requirements differ if the material being tested is a new chemical entity or an established product?

(continued)

Country	Answer
Ireland	N/A
Norway	N/A
Lithuania	No - the requirements are the same.
Switzerland	We have not had requests for such interventions.

5. Does your national agency have any requirements for what data should be provided and/or the format in which the data should be provided that is derived from human sensory evaluation studies?

Country	Answer
Croatia	Agency does not have any specific national requirements for what data should be provided neither the format in which the data should be provided.
Czech Republic	No specific requirements.
Estonia	N/A
Germany	No.
Italy	No specific requirements so far (see Q4).
Poland	No.
Romania	No.
Slovenia	We haven't received any information on such studies so far.
Sweden	We do not have any specific requirements and refers to the information given in the paediatric guideline, Guideline on pharmaceutical development of medicines for paediatric use regarding acceptability studies to be performed in the paediatric population.
UK	The requirements for a palatability study are stated in a "Guideline on pharmaceutical development of medicines for paediatric use (EMA/CHMP/QWP/805880/2012 Rev. 2). There is no specific requirement for the type or format of the data presented; however, this is expected to include: a clear description of the study procedures and study population, description of the test products, description of any statistics applied, description of study outcomes per test subject, an overall conclusion.

(continued)

5. Does your national agency have any requirements for what data should be provided and/or the format in which the data should be provided that is derived from human sensory evaluation studies?

(continued)

Country	Answer
Ireland	We have no specific requirements outside that which would be seen in any typical clinical trial report.
Norway	The NoMA does not have any specific national requirements on human sensory evaluation studies. It is expected that any EMA guidelines are adhered to and if necessary collaboration with other NCAs on specific topics is considered.
Lithuania	No specific local requirements.
Switzerland	We have not had requests for such interventions.

6. Would your national agency consider data from palatability assessments performed with healthy adult volunteers‘ panel adequate data to support evidence of palatability in the targeted population (e.g. paediatric) in the drug development dossier?

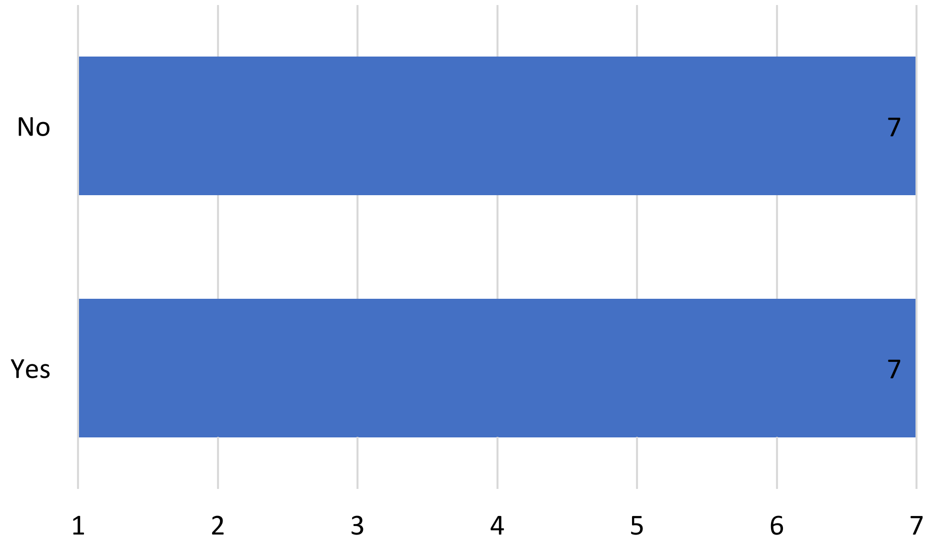

Estonia, Germany, Ireland, Romania, Lithuania, Norway, Slovenia

Croatia, Czech Republic Italy, Poland, Sweden, UK, Switzerland

Answer	%	Count
Yes	50%	7
No	50%	7
Total	100%	14

Explanations to Q6

Country	Explanation
Croatia	Yes, studies in dedicated adults panels or relevant literature references would be acceptable.
Czech Republic	Depending on case by case basis (depending e.g. on the age of children).
Estonia	Results from palatability studies can be very different in adults and children.
Germany	Data should be collected from the target population.
Italy	Case by case approach is recommended depending on the medication (e.g. chemotherapy agents) and clinical setting.
Poland	Every additional data which help set safe boundaries for children is Good clinical practice.
Romania	Generation of palatability data is required to be assessed in the target population.
Slovenia	We would apply the same conditions as for the paediatric population in clinical trials.
Sweden	In accordance with the Guideline on pharmaceutical development of medicines for paediatric use this could be accepted in justified cases. This is if no clinical studies will be conducted in children or where patient acceptability will not be studied as part of the paediatric clinical studies, adequate patient acceptability of the medicine(s) as proposed for marketing could be demonstrated by other means e.g. by literature references or by studies in dedicated adult panels.
UK	Yes, depending on the type of application, adult data would be acceptable.

(continued)

Explanations to Q6

(continued)

Country	Explanation
Ireland	Supportive and indicative, but not pivotal.
Norway	Palatability assessment data from adults would be considered with justifications on how it relates to expected target population. Challenges regarding palatability studies in paediatric populations can justify using an adult panel. This is in line with a guideline on medicines for the geriatric population which could be applied here as well. “Adequate patient acceptability can be demonstrated by different means (e.g. using data from clinical trials, representative simulated use studies, human factor studies with healthy volunteers or patients, market experiences, literature). As knowledge on testing a product’s acceptability in the older patient population is fragmented, the selection of the method and acceptance criteria is left to the company. However, companies will need to justify their approach (e.g. design of the trial, limit(s)) with respect to the product benefit to risk considerations in each of the relevant subsets of the older population. This justification would include consideration of the risk of poor adherence, any alternative administration strategies and medication errors.”
Lithuania	EMA guidance states: Evaluation of the patient acceptability of a paediatric preparation should be an integral part of the pharmaceutical and clinical development. Patient acceptability of a preparation should preferably be studied in children themselves as part of a clinical study involving the proposed medicinal product (page 19). It will considered on case-by-case basis.

Explanations to Q6

(continued)

Country	Explanation
Switzerland	We will review such requests and act accordingly.

7. What evidence would your national agency ask for if a sponsor wished to make a claim about the sensory aspects of their product (for example that the product has superior palatability to a previous formulation)?

Country	Answer
Croatia	superior palatability to a previous formulation.
Czech Republic	Avoiding such claim is preferred (depending on case by case basis), it is perceived as marketing claim.
Estonia	N/A
Germany	It would be expected that the formulation is acceptable to children, meaning that the AM can be administered without major problems.
Italy	N/A
Poland	Depend on drug.
Romania	Generation of the palatability data with the new formulation comparative with that one using the previous formulation.
Slovenia	We don`t have experience on this issue.
Sweden	There has to be a comparable study performed in the target age groups showing a statistical relevant benefit for the formulation claimed to be superior.
UK	See above for data requirements under Q5.

(continued)

7. What evidence would your national agency ask for if a sponsor wished to make a claim about the sensory aspects of their product (for example that the product has superior palatability to a previous formulation)?

(continued)

Country	Answer
Ireland	We have not formulated specific guidance on this, so would assess any such application on its own merits.
Norway	Claims of sensory aspect should be supported with data. As highlighted in the guideline referenced above it is the companies responsibility of choosing a suitable method to demonstrate this and provide justification for the chosen method.
Lithuania	Clinical data.
Switzerland	We have not had such requests at the moment.

8. Would a similar approach be taken for other non-oral organoleptic sensory studies e.g. for topical/transdermal products, nasal/inhaled products?

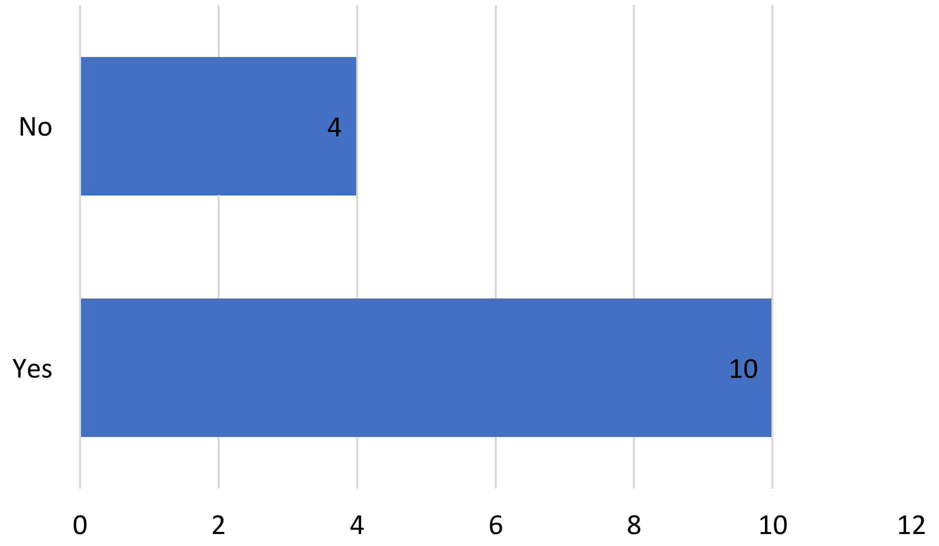

Estonia, Germany, Ireland, Switzerland

Croatia, Czech Republic Italy, Norway, Poland, Romania, Slovenia, Sweden, UK, Lithuania

Answer	%	Count
Yes	71%	10
No	29%	4
Total	100%	14

Explanation to Q8

Country	Explanation
Croatia	N/A
Czech Republic	bud depending on case by case basis.
Estonia	N/A
Germany	For non-oral products acceptability and tolerability should be tested.
Italy	N/A
Poland	Every studies need to set by NCA and GCP standards.
Romania	Similar approach for topical/transdermal products, nasal/inhaled products is encouraged as this would play an important role in the acceptability of the product.
Slovenia	N/A
Sweden	N/A
UK	As stated in Q5 there are no specific requirements so data relating to other sensory studies would be considered. It should be note that “Guideline on pharmaceutical development of medicines for paediatric use (EMA/CHMP/QWP/805880/2012 Rev. 2)” specifically mentions the palatability of products in relation to “nasal administration or inhalation”.

(continued)

Explanation to Q8

(continued)

Country	Explanation
Ireland	I believe there are more specific guidance documents available for the assessment of such products.
Norway	It is considered the company's responsibility to ensure adequate data is presented on relevant properties of the product.
Lithuania	It will depend on the particular situation and product.
Switzerland	We have not had request for such in other non-oral studies: topical/transdermal products, nasal/inhaled products?

9. Finally, some sensory data is available using non-human studies, for example e-tongue and rodent BATA (brief access taste aversion) studies. Does your agency accept such data as evidence of the palatability of formulations?

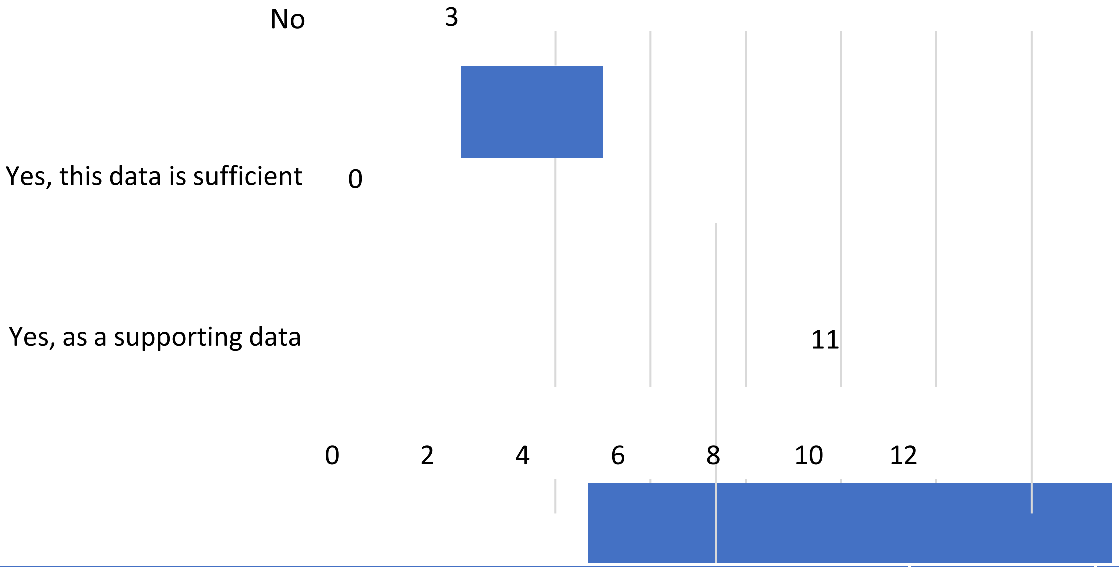

Estonia, Romania, Switzerland

Croatia, Czech Republic, Germany, Ireland, Italy, Norway, Poland, Slovenia, Sweden, UK, Lithuania

Answer	%	Count
Yes, this data is sufficient on its own	0%	0
Yes, as supporting data to human panel data	79%	11
No	21%	3
Total	100%	14

Explanation to Q9

Country	Explanation
Croatia	N/A
Czech Republic	N/A
Estonia	N/A
Germany	Animal data would be accepted as supportive.
Italy	Likely they can be accepted as supportive data.
Poland	N/A
Romania	To my knowledge no such data have been accepted as evidence for the assessment of the palatability in the drug development dossier.
Slovenia	In our opinion this could be supportive data, however we have no experience on this issue.
Sweden	N/A
UK	Depending on the type of application, these data may be supportive or sufficient of their own, assuming the validation of the test can be demonstrated. In some cases non-human studies are encountered early in development screening out several formulations. The palatability of the final formulation(s) can later be confirmed in humans.

(continued)

Explanation to Q9

(continued)

Country	Explanation
Ireland	No specific guidance exists, but this would not appear sufficient in itself.
Norway	None of the above necessarily true: All data would be considered. But data will need to be justified and it relevance to the target population explained. This is in line with the guideline referenced above.
Lithuania	N/A
Switzerland	We have not received such requests and so difficult to give an opinion here.

10. In any sensory study there will be a proportion of respondents who find the product to be palatable and a proportion that do not. Does your agency have any guidance on the overall proportion of respondents that must find the product to be palatable for it to be approved?

Country	Answer
Croatia	Croatian agency does not have guidance on the overall proportion of respondents that must find the product to be palatable.
Czech Republic	No.
Estonia	N/A
Germany	No.
Italy	N/A
Poland	Every case will be treated individual.
Romania	No specific guideline for the assessment of the palatability.
Slovenia	Currently, we have no guidelines.
Sweden	At the time being we do not have any guidance for this issue, but this could be considered at assessment of the study results, if acceptably discussed and justified by the sponsor.
UK	No, we do not apply a common threshold approach across studies/products. The evaluation will e.g. depend on the type of product, the test outcome for comparators used, the response scale used etc.

(continued)

10. In any sensory study there will be a proportion of respondents who find the product to be palatable and a proportion that do not. Does your agency have any guidance on the overall proportion of respondents that must find the product to be palatable for it to be approved?

(continued)

Country	Answer
Ireland	No specific guidance exists, but it would seem that any "success" proportion should be greater than 80%, although it is accepted that this is arbitrary.
Norway	There is no internal guideline available on the proportion of respondents to establish an outcome of a study. This would need to be considered along with the testing group, tested parameters, relevance to target population etc. As such, decisions would be performed on case by case.
Lithuania	No specific guidance.
Switzerland	We have not yet developed such guidance in the clinical trials division.
